# European Lepidoptera–Plant Associations: A Species‐Level Interaction Matrix

**DOI:** 10.1002/ece3.73004

**Published:** 2026-02-15

**Authors:** Álvaro Gaytán

**Affiliations:** ^1^ Institute of Natural Resources and Agrobiology of Seville (IRNAS‐CSIC) Seville Spain

**Keywords:** biodiversity data, ecological networks, herbivores, host specialization, Lepidoptera‐plant interactions, resource database

## Abstract

Documenting plant–herbivore interactions at broad spatial scales is crucial for understanding the ecological and evolutionary processes that shape biodiversity. Here I present a continental‐scale interaction matrix encompassing 5152 Lepidoptera species and 3275 vascular plant species across Europe. The dataset compiles records from published literature, monographs, online repositories, and gray sources, with all taxa standardized to current classifications. By resolving associations at the species level, this resource provides a robust basis for investigating host specialization, network structure, and patterns of herbivore diversity in biogeographic and phylogenetic contexts. In addition to supporting fundamental research, the matrix offers applied value for conservation biology—for example, by identifying keystone host plants that sustain diverse herbivore assemblages and by assessing the vulnerability of insect communities to host loss under global change. The dataset is openly available in a machine‐readable format under a CC‐BY 4.0 license, ensuring transparency, reproducibility, and long‐term accessibility for ecological and evolutionary studies.

## Abstract 2

A continental‐scale interaction matrix encompassing 5152 Lepidoptera species and 3275 vascular plant species across Europe.

## 1 Introduction

Plant–herbivore interactions are fundamental drivers of terrestrial ecosystems, shaping plant fitness, community assembly, and the flow of energy across trophic levels (Price 1997). Within this context, Lepidoptera stands out as one of the most diverse and ecologically significant groups of herbivorous insects, with larval stages exploiting a vast array of host plants (Forister et al. 2015). Owing to their ecological roles and pronounced sensitivity to environmental change, Lepidoptera are widely employed as model systems in ecology, evolution, and conservation biology (Bale et al. 2002; Narango et al. 2020; Gaytán et al. 2020, 2022). Despite this importance, comprehensive resources that systematically document Lepidoptera–host plant associations at broad spatial scales remain scarce. Existing databases provide valuable insights (Robinson et al. 2023; Ellis 2025; Jonko 2025), yet they often lack the combination of species‐level resolution, rigorous taxonomic curation, and open accessibility needed for large‐scale comparative research.

**FIGURE 1:**
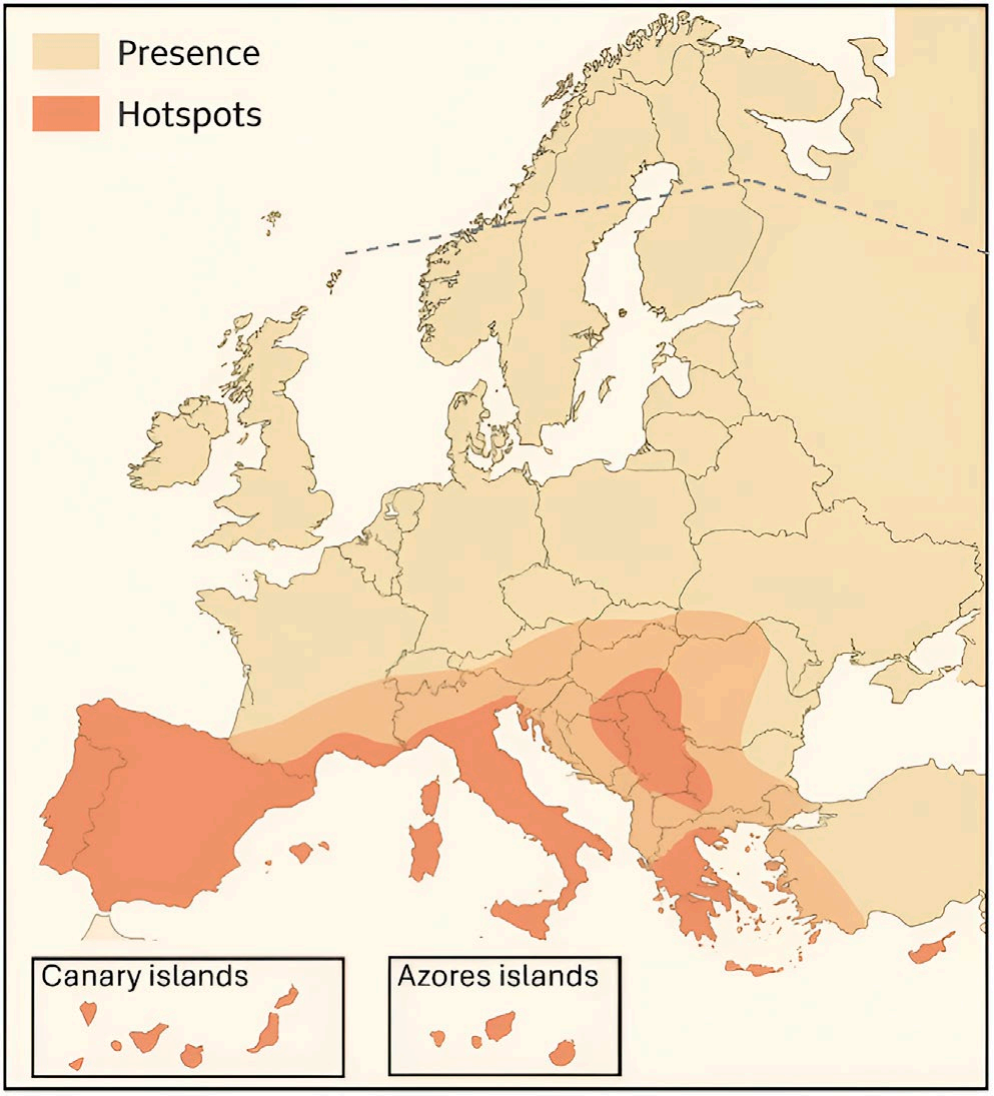
Limit of distribution of Lepidoptera in Europe. Shaded areas indicate the overall presence of lepidopterans across Europe, with darker tones highlighting major hotspots of species richness and endemism. Insets show the Canary Islands and the Azores. The dashed northern boundary marks indicate the climatic transition where Lepidoptera species richness drops markedly toward the Arctic.

**FIGURE 2:**
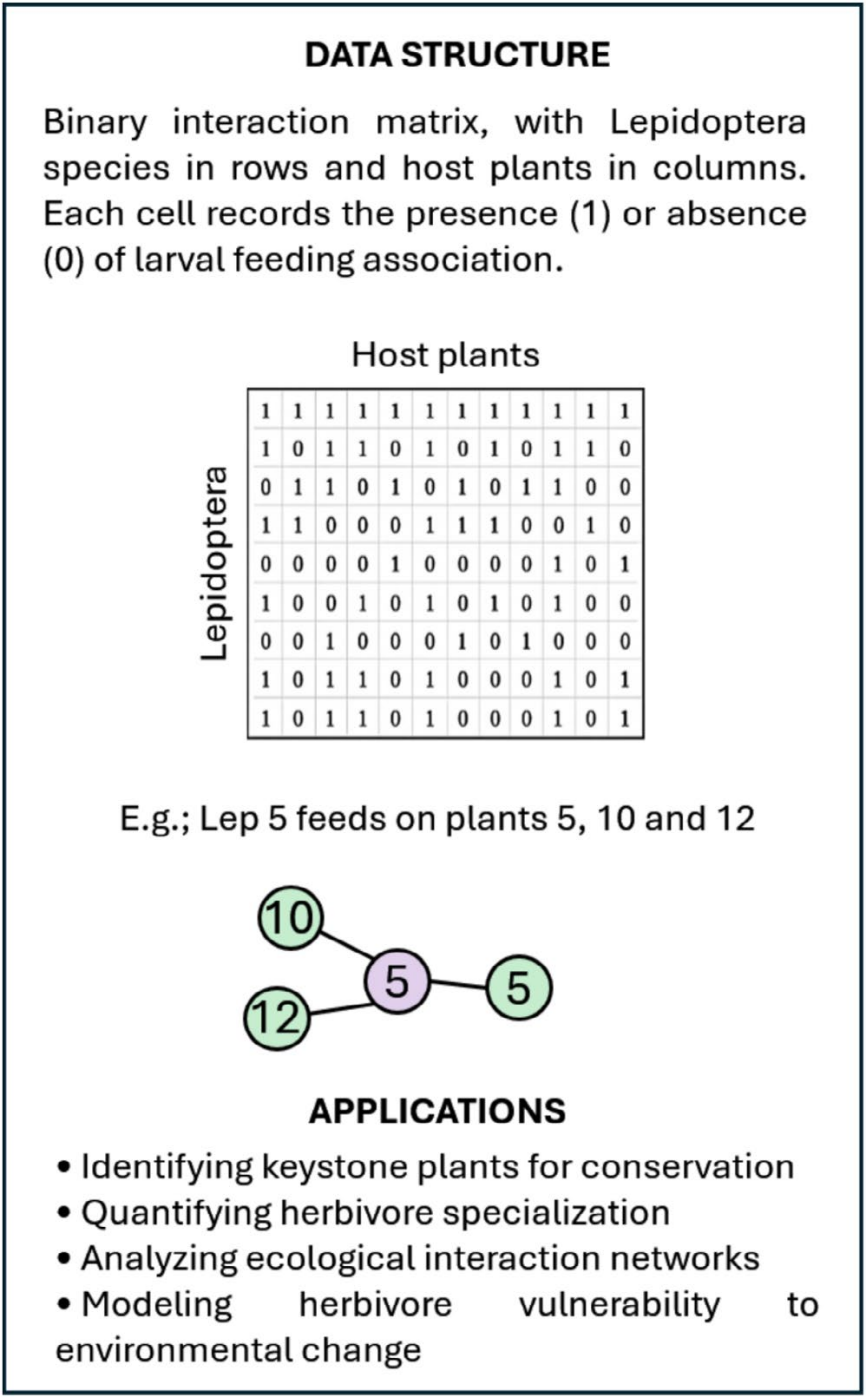
Conceptual overview of the Lepidoptera–plant interaction matrix.

Here, I introduce a continental‐scale interaction matrix that compiles larval feeding associations between European Lepidoptera and vascular plants (Figure 1). The dataset encompasses more than 5100 herbivore species and over 3200 host plants, making it one of the most extensive standardized resources of its kind. By integrating taxonomic precision with broad coverage, this matrix provides a robust foundation for investigating host specialization, interaction network structure, and patterns of herbivore diversity, while also supporting applied research in biodiversity conservation and ecosystem management (Figure 2).

## 2 Methods

The dataset is structured as a binary interaction matrix, where rows correspond to Lepidoptera species and columns to vascular plant species. Each cell records the presence (1) or absence (0) of a larval feeding association (Figure 2). In total, the matrix documents 5109 Lepidoptera species and 3175 host plants, stored in a comma‐separated values (.CSV) file of 32.6 MB. Column headers are standardized scientific names of plants, while row labels represent lepidopteran taxa. The dataset contains no missing values, and all entries are coded as integers (0/1).

Associations were compiled through an extensive review of published monographs, primary literature, gray sources, and open‐access biodiversity repositories (see Appendix S1 for the final curated list of references used to construct this matrix). To ensure consistency, all taxonomic names were curated and cross‐validated against authoritative references, including Robinson et al. (2023), Cerdeira‐Pérez et al. (2025), Ellis (2025), Euro+Med (2025), IPNI (2025), Jonko (2025), and WFO (2025). Synonyms and outdated nomenclature were harmonized following the most recent accepted classifications.

Quality control involved three steps: (i) duplicate records across sources were identified and merged, (ii) taxonomic inconsistencies were flagged and corrected using the aforementioned references, and (iii) all binary transformations were validated by random subsampling and manual verification against original sources. Because the dataset is standardized at the species level and integrates native, naturalized, ornamental, and invasive plants, it provides a comprehensive representation of interactions relevant to both ecological research and applied conservation. Alongside the matrix, I added a vector classifying each plant species in the dataset into the eight most common European ecosystems (boreal coniferous, boreal tundra, continental temperate, grasslands, islands, Mediterranean, mountain, oceanic temperate), with an additional category for non‐native species. I classified each Lepidoptera species as native or non‐native to Europe to contextualize interactions involving non‐native plants and to clarify the biogeographic scope of the dataset.

## 3 Discussion and Applications

This dataset constitutes one of the most comprehensive resources on Lepidoptera–plant interactions available at a continental scale (Table S1). Its breadth and species‐level resolution open avenues for addressing fundamental ecological and evolutionary questions, including the prevalence and drivers of host specialization, the determinants of herbivore diversity, and the structure of plant–herbivore networks across Europe. By resolving associations at fine taxonomic detail, the matrix also enables analyses of niche partitioning in relation to host traits, plant phylogeny, and biogeographic context.

In addition to its value for basic research, the dataset has strong applied relevance. It can help identify keystone host plants supporting disproportionate numbers of Lepidoptera species, assess the vulnerability of herbivore assemblages to host loss under land‐use change, agricultural intensification, or climate change, and guide conservation strategies targeting both insect and plant diversity. Integration with environmental, phylogenetic, or trait‐based datasets further enhances its potential for predictive modeling, risk assessment, and long‐term biodiversity monitoring.

By releasing the resource in a machine‐readable format, with detailed metadata and open licensing (CC‐BY 4.0), I aim to maximize transparency, reproducibility, and interoperability with other biodiversity databases. Free accessibility is intended to foster broad engagement across ecological and conservation communities, strengthen comparative studies of plant–herbivore interactions, and provide a lasting foundation for research on the diversity, specialization, and resilience of European herbivore assemblages. This contribution lacks detailed geographical information, reflecting a limitation inherent to the data source and the difficulty of accurately mapping the distribution of so many species. Users should not assume that plant–lepidoptera interactions are geographically invariant, as they may vary with factors such as plant distribution and climate. This dataset is designed as a dynamic resource. Future updates will progressively incorporate ecological context and functional traits, further expanding its utility for both fundamental research and applied conservation.

## 4 Dataset Overview

Associations are organized as a binary interaction matrix, where rows represent lepidopterans and columns correspond to plant species. Each cell contains a value of 1 to indicate a recorded association or 0 to denote the absence of a record. Geographic coverage adheres to the boundaries defined by the European Environment Agency. Taxonomic names were standardized using current classifications for both Lepidoptera and vascular plants. The dataset is available in CSV format (UTF‐8 encoded), with standardized scientific names used for all row and column headers. Three files are provided: the main data matrix, a file with the taxonomic classification of each lepidoptera and plant species (Taxonomy.xlsx) and a secondary matrix where all plant species are classified in the main European ecosystem (Ecosystem.csv), together with a README.md detailing the dataset structure and usage guidelines. The dataset is released under a Creative Commons Attribution 4.0 License (CC‐BY 4.0), allowing reuse, redistribution, and adaptation with appropriate credit to the original authors.

## Author Contributions

**Álvaro Gaytán:** conceptualization (equal), data curation (equal), investigation (equal), methodology (equal), supervision (equal), validation (equal), writing – original draft (equal), writing – review and editing (equal).

## Conflicts of Interest

The author declares no conflicts of interest.

## Supporting information


**Appendix S1:** List of references to compile plant‐lepidoptera associations.
**Table S1:** Summary of taxonomic composition by family for lepidoptera and host plants, where number of species in each family (#) and the percentage of species out the total number of species of each group (%) is shown.

## Data Availability

All data associated with this publication are openly available through the Environmental Data Initiative (EDI) under the Creative Commons Attribution 4.0 International License (CC BY 4.0). The dataset can be accessed at Zenodo via the following DOI: 10.5281/zenodo.16914288. All metadata follow the Ecological Metadata Language (EML) standard to ensure interoperability and long‐term accessibility. I support open science practices and encourage the reuse of these materials for research, education, and conservation planning. Users are requested to cite this paper when using the data.
