# Supplementary material for: European Lepidoptera–Plant Associations: A Species‐Level Interaction Matrix
Source: Ecol Evol. 2026 Feb 15;16(2):e73004. doi: 10.1002/ece3.73004 (PMC12906973; doi:10.1002/ece3.73004)
Supplement: Supplementary file 1 — Appendix S1: List of references to compile plant‐lepidoptera associations. Table S1: Summary of taxonomic composition by family for lepidoptera and host plants, where number of species in each family (#) and the percentage of species out the total number of species of each group (%) is shown. [file ECE3-16-e73004-s001.docx]

**Appendix 1.** List of references to compile plant-lepidoptera associations

1. Aarvik, L., Bengtsson, B. Å., Elven, H., Ivinskis, P., Jürivete, U., Karsholt, O., Mutanen, M., & Savenkov, N. (2023). Nordic-Baltic Lepidoptera Database (NOBALIS). <https://www.nobalis.org>
2. Aguiar, A. M., & Karsholt, O. (2020). Non-native insect pests from the Madeira Archipelago (Portugal): New records and further data. Fragmenta Entomologica, 52(2), 147–162. https://doi.org/10.13133/2284-4880/469
3. Aistleitner, E. (2010). Catalogue of the Lepidoptera of Austria. Entomological Society of Austria.
4. Anton, C., Musche, M., Hula, V., & Settele, J. (2007). Interactions of climate and habitat quality on the population dynamics of the endangered butterfly Maculinea nausithous. Oecologia, 153(3), 545–557.
5. Asher, J., Warren, M., Fox, R., Harding, P., Jeffcoate, G., & Jeffcoate, S. (2001). The Millennium Atlas of Butterflies in Britain and Ireland. Oxford University Press.
6. Balletto, E., & Kudrna, O. (1985). Ecology and biogeography of European butterflies. Monographs on Lepidoptera, 1, 1–250.
7. Barbour, D. A., & Oates, M. R. (2015). The conservation of European butterflies: Managing host plants and habitats. Journal of Insect Conservation, 19(4), 621–635.
8. Bellmann, H. (2011). Guide to the Butterflies of Europe: Butterflies, caterpillars and host plants. Kosmos.
9. Beneš, J., Konvička, M., Dvořák, J., Fric, Z., Havelda, Z., Pavlíčko, A., … Vrabec, V. (2002). Butterflies of the Czech Republic: Distribution and conservation I–II. SOMS Press.
10. Bertaccini, E., Parenzan, P., & Dapporto, L. (2015). Butterflies of Italy: Systematics, ecology and distribution. Edizioni Belvedere.
11. Betzholtz, P. E., & Franzén, M. (2013). Host plant availability drives landscape distribution of threatened butterflies in southern Sweden. Insect Conservation and Diversity, 6(6), 639–650.
12. Betzholtz, P. E., Pettersson, L. B., Ryrholm, N., & Franzén, M. (2013). With that diet, you will go far: Trait-based analysis reveals links between larval host plants and butterfly distributional patterns in Europe. Ecography, 36(5), 529–539.
13. Bink, F. A. (1992). Ecological atlas of the butterflies of the Netherlands. Schuyt & Co.
14. Bladmineerders.nl (Ellis, W. N.). (2025). Plant Parasites of Europe: Leafminers, galls and fungi. <https://bladmineerders.nl>
15. Böhme, J. (2001). The butterflies and moths of Central Europe. Quelle & Meyer Verlag.
16. Borges, P. A. V., Costa, A., Cunha, R., Gabriel, R., Gonçalves, V., Martins, A. F., Melo, I., Parente, M., Raposeiro, P., Rodrigues, P., Santos, R. S., Silva, L., Vieira, P., & Vieira, V. (Eds.). (2010). A list of the terrestrial and marine biota from the Azores. Princípia.
17. Bryk, F. (1948). Lepidoptera of the Arctic and Northern Europe. Acta Entomologica Fennica, 5, 1–240.
18. Buszko, J. (2024). Lepidoptera.pl: Butterflies and moths of Poland. <https://www.lepidoptera.pl>
19. Cerdeira-Pérez, A., Laanisto, L., & Puglielli, G. (2025). InsectGUILD: Feeding guilds of lepidopteran and hymenopteran larvae consuming Northern Hemisphere woody plants. Scientific Data, 12(1), 887. <https://doi.org/10.1038/s41597-025-02349-8>
20. Cizek, L., Fric, Z., & Konvicka, M. (2006). Host plant and habitat use of the declining butterfly Euphydryas aurinia in the Czech Republic. European Journal of Entomology, 103, 675–681.
21. Cizek, O., Fric, Z., & Konvicka, M. (2011). Host specialization and conservation of Maculinea butterflies in fragmented landscapes. Ecological Entomology, 36(6), 641–653.
22. Clemens, R., & Hausmann, A. (2023). Lepiforum e.V. – Bestimmungshilfe für die europäischen Schmetterlinge. <https://lepiforum.org>
23. Clarke, H. E. (2022). A provisional checklist of European butterfly larval foodplants. Nota Lepidopterologica, 45, 139–167. <https://doi.org/10.3897/nl.45.72017>
24. Clarke, H. E. (2024). A checklist of European butterfly larval foodplants. Ecology and Evolution, 14(2), e11039. <https://doi.org/10.1002/ece3.11039>
25. Dierks, A., & Fischer, K. (2009). Feeding behaviour and resource use of European burnet moths (Zygaenidae). Journal of Insect Physiology, 55(7), 658–665.
26. Dincă, V., Dapporto, L., & Vila, R. (2011). A combined genetic–morphometric analysis reveals new cryptic butterfly species in the European fauna. Biological Journal of the Linnean Society, 103(4), 831–845.
27. Descimon, H., & Mallet, J. (2009). Bad species. In J. Settele, T. Shreeve, M. Konvicka, & H. Van Dyck (Eds.), Ecology of Butterflies in Europe (pp. 219–249). Cambridge University Press.
28. Descimon, H., Bachelard, P., Boitier, E., & Lafranchis, T. (2005). Decline and conservation of butterflies in France: Patterns, causes and conservation implications. Biodiversity and Conservation, 14(1), 131–152.
29. Ebert, G. (Ed.). (2003). Die Schmetterlinge Baden-Württembergs. Vol. 10: Supplement and host plant index. Ulmer Verlag.
30. Ebert, G. (1997). Die Schmetterlinge Baden-Württembergs. Vol. 8: Nachtfalter VI. Ulmer Verlag.
31. Ebert, G., & Rennwald, E. (1991). Die Schmetterlinge Baden-Württembergs. Ulmer Verlag.
32. Eitschberger, U. (2013). Sphingidae: Hawk moths of Europe. Selbstverlag Eitschberger.
33. Eliasson, C. U. (2012). Butterflies and Moths of Scandinavia: Ecology, Distribution and Host Plants. Swedish Entomological Society.
34. Eliasson, C. U., Ryrholm, N., & Gärdenfors, U. (2005). Nationalnyckeln till Sveriges flora och fauna: Fjärilar: Dagfjärilar: Hesperiidae–Nymphalidae. Swedish Species Information Centre.
35. Emmet, A. M., & Heath, J. (Eds.). (1989–2002). The Moths and Butterflies of Great Britain and Ireland (Vols. 1–11). Harley Books.
36. European Environment Agency (EEA). (2023). Butterfly Indicator for Europe. <https://www.eea.europa.eu/data-and-maps/indicators/grassland-butterflies-2>
37. Fauna Europaea. (2025). Taxonomic Index of European Animals: Lepidoptera. <https://fauna-eu.org>
38. Forister, M. L., Novotny, V., Panorska, A. K., Baje, L., Basset, Y., Butterill, P. T., et al. (2015). The global distribution of diet breadth in insect herbivores. Proceedings of the National Academy of Sciences, 112(2), 442–447.
39. Fric, Z., & Konvicka, M. (2002). Host plant and habitat preferences of the endangered butterfly Euphydryas maturna in the Czech Republic. Acta Entomologica Bohemoslovaca, 99(3), 253–265.
40. García-Barros, E., Munguira, M. L., Stefanescu, C., & Vives Moreno, A. (2004). Atlas of the Butterflies of the Iberian Peninsula and Balearic Islands (Lepidoptera: Papilionoidea & Hesperioidea). SEA Publications.
41. Goffart, P. (2014). Les Papillons de jour de Wallonie et de Bruxelles. SPW Éditions.
42. Habel, J. C., Rödder, D., & Schmitt, T. (2011). Climate change and butterfly range dynamics: Lessons from Europe. Biodiversity and Conservation, 20(4), 729–741.
43. Hacker, H., & Fibiger, M. (2007). Noctuidae Europaeae. Volume 9: Amphipyrinae, Condicinae, and Eriopinae. Entomological Press.
44. Harvey, D. J., Warren, M. S., & Harding, P. (2012). Atlas of Butterflies in Britain and Ireland: Ecology and host plants. Butterfly Conservation Trust.
45. Hausmann, A. (2001). The Geometrid Moths of Europe. Volume 1: Introduction and Sterrhinae. Apollo Books.
46. Hausmann, A. (Ed.). (2004). The Geometrid Moths of Europe. Volume 2: Larentiinae II. Apollo Books.
47. Hausmann, A., & Scalercio, S. (2016). Host-plant relationships of 29 Mediterranean Lepidoptera species in forested ecosystems unveiled by DNA barcoding. SHILAP Revista de Lepidopterología, 44(173), 99–115.
48. Hernández-Teixidor, D., Santos, I., Suárez, D., & Oromí, P. (2020). The importance of threatened host plants for arthropod diversity: The fauna associated with dendroid Euphorbia plants endemic to the Canary and Madeira archipelagos. Journal of Insect Conservation, 24, 867–876. https://doi.org/10.1007/s10841-020-00261-z
49. Hesselbarth, G., van Oorschot, H., & Wagener, S. (1995). Die Tagfalter der Türkei unter Berücksichtigung der angrenzenden Länder. Selbstverlag.
50. Jonko, C. (2025). Lepidoptera mundi: Online guide to the world butterflies and moths. <https://lepidoptera.eu/start>
51. Karsholt, O., & Razowski, J. (Eds.). (1996). The Lepidoptera of Europe: A Checklist. Apollo Books.
52. Karsholt, O., Mutanen, M., & Aarvik, L. (2013). Fauna Europaea: Lepidoptera. Biodiversity Data Journal, 1, e102.
53. Konvicka, M., Cizek, O., Beneš, J., Kopeček, F., & Němec, T. (2008). Habitat and host plant preferences of endangered European butterflies. Journal of Insect Conservation, 12, 527–538.
54. Kudrna, O., Harpke, A., Lux, K., Pennerstorfer, J., Schweiger, O., Settele, J., & Wiemers, M. (2015). Distribution Atlas of European Butterflies and Skippers. Wissenschaftlicher Verlag.
55. Lafranchis, T. (2004). Butterflies of Europe. Diatheo.
56. Lafranchis, T. (2019). Les Papillons de jour de France, Belgique et Luxembourg et leurs chenilles. Diatheo.
57. Lafranchis, T., & van der Poorten, D. (2015). The Butterflies of Europe and their Ecology. Diatheo.
58. Little Oak Group (2023). Azores wildlife: birds, cetaceans & other wildlife. URL: [www.azoreswildlife.com](http://www.azoreswildlife.com)
59. Loertscher, M., Benz, G., & Erhardt, A. (1995). Importance of host plant abundance for population structure of the scarce copper Lycaena virgaureae. Ecological Entomology, 20(3), 309–316.
60. Mazzei, P., Reggianti, D., & Pimpinelli, I. (2005–2025). Moths and Butterflies of Europe and North Africa. <https://www.leps.it>
61. Munguira, M. L., & Martín, J. (1999). Action plan for the Maculinea butterflies in Europe. Nature and Environment Series, 97. Council of Europe Publishing.
62. Nádenik, J., Beneš, J., & Konvicka, M. (2007). Larval feeding ecology of endangered European butterflies: A case study of Euphydryas aurinia. Journal of Insect Conservation, 11(2), 173–182.
63. Newland, D., Still, R., & Swash, A. (2015). Britain’s Butterflies: A field guide to the butterflies of Britain and Ireland. Princeton University Press.
64. Niehuis, M., & Naumann, C. M. (2006). Phylogenetic relationships and host plant evolution in Zygaenidae (Lepidoptera). Biological Journal of the Linnean Society, 87(2), 145–156.
65. Parenzan, P., & Porcelli, F. (2006). Lepidoptera of Italy: A comprehensive checklist. Edizioni Belvedere.
66. Popescu-Gorj, A. (1986). Catalogul Lepidopterelor României. Editura Academiei Române.
67. Puplesis, R., & Robinson, G. S. (2000). The Nepticulidae and Opostegidae (Lepidoptera) of North and West Europe. Apollo Books.
68. Rákosy, L., & Goia, M. (2007). Butterflies of Romania: Distribution and ecology. Cluj University Press.
69. Robinson, G. S., Ackery, P. R., Kitching, I. J., Beccaloni, G. W., & Hernández, L. M. (2010). HOSTS — A database of the world’s lepidopteran host plants. Natural History Museum, London. <http://www.nhm.ac.uk/hosts>
70. Ryrholm, N. (2003). Butterflies and climate change in Northern Europe. Entomologisk Tidskrift, 124, 133–144.
71. Saarinen, K., Lahti, T., & Marttila, O. (2003). Habitat associations and host plant use of Finnish butterflies. Annales Zoologici Fennici, 40, 133–146.
72. Seifert, C. L., Kallioniemi, E., & Gossner, M. M. (2024). Macroecological patterns in European butterflies unveil strong constraints from host plant affinities. Ecography, 47(3), e07021.
73. Seifert, C. L., Settele, J., & Schweiger, O. (2023). Host plant use and seasonal life cycles in European geometrid moths. Journal of Animal Ecology, 92(4), 743–755.
74. Settele, J., Shreeve, T., Konvicka, M., & Van Dyck, H. (Eds.). (2009). Ecology of Butterflies in Europe. Cambridge University Press.
75. Sonderegger, P. (2005). Die Erebidae und Noctuidae der Schweiz: Verbreitung, Ökologie und Raupennahrungspflanzen. Entomologische Gesellschaft Zürich.
76. Stefanescu, C., Penuelas, J., & Filella, I. (2009). Rapid changes in butterfly communities following drought events in Mediterranean habitats: Role of host plant availability. Global Change Biology, 15(3), 1217–1230.
77. Tennent, J. (2015). The Butterflies of Britain and Ireland (3rd ed.). British Wildlife Publishing.
78. Tolman, T., & Lewington, R. (2008). Collins Butterfly Guide: The most complete guide to the butterflies of Britain and Europe. HarperCollins.
79. Tolman, T., & Lewington, R. (2009). The Collins Field Guide to the Butterflies of Britain and Europe. HarperCollins.
80. Tshikolovets, V. V. (2011). Butterflies of Europe and the Mediterranean Area. Tshikolovets Publications.
81. Wahlberg, N., & Viidalepp, J. (2000). Phylogeny and host plant relationships of the tribe Macariini (Geometridae: Ennominae). Systematic Entomology, 25(3), 289–301.
82. Wagner, W. (2025). Pyrgus.de — Lepidoptera and their ecology. <https://www.pyrgus.de>
83. Wiemers, M., Chazot, N., Wheat, C. W., Schweiger, O., Wahlberg, N., & Lamas, G. (2018). An updated checklist of the European butterflies (Lepidoptera, Papilionoidea). ZooKeys, 811, 9–45.
84. Ziegler, H. (2024). Euroleps.ch: Butterflies and Moths of Europe. <https://www.euroleps.ch>
85. Zobodat. (2025). Biodiversity database of Austria: Lepidoptera distribution and host records. <https://www.zobodat.at>
86. Zygaena Database (Niehuis, M., & Naumann, C. M.). (2023). Host plant data and taxonomy of European burnet moths. <https://www.zygaenidae.de>

**Table S1.** Summary of taxonomic composition by family for lepidoptera and host plants, where number of species in each family (#) and the percentage of species out the total number of species of each group (%) is shown.

| **Lepidoptera family** | **#** | **%** | **Plant family** | **#** | **%** |
| --- | --- | --- | --- | --- | --- |
| Acrolepiidae | 17 | 0.33 | Acanthaceae | 3 | 0.09 |
| Adelidae | 25 | 0.49 | Acoraceae | 1 | 0.03 |
| Alucitidae | 10 | 0.19 | Actinidiaceae | 2 | 0.06 |
| Autostichidae | 1 | 0.02 | Adoxaceae | 8 | 0.25 |
| Batrachedridae | 2 | 0.04 | Aizoaceae | 1 | 0.03 |
| Bedelliidae | 1 | 0.02 | Alismataceae | 1 | 0.03 |
| Bistoninae | 2 | 0.04 | Altingiaceae | 1 | 0.03 |
| Blastobasidae | 8 | 0.16 | Amaranthaceae | 82 | 2.57 |
| Bombycidae | 2 | 0.04 | Amaryllidaceae | 18 | 0.56 |
| Brachodidae | 4 | 0.08 | Anacardiaceae | 16 | 0.50 |
| Brahmaeidae | 7 | 0.14 | Apiaceae | 125 | 3.92 |
| Bucculatricidae | 49 | 0.95 | Apocynaceae | 22 | 0.69 |
| Carposinidae | 2 | 0.04 | Aquifoliaceae | 2 | 0.06 |
| Castniidae | 1 | 0.02 | Araceae | 8 | 0.25 |
| Choreutidae | 14 | 0.27 | Araliaceae | 2 | 0.06 |
| Coleophoridae | 237 | 4.60 | Arecaceae | 4 | 0.13 |
| Cosmopterigidae | 24 | 0.47 | Aristolochiaceae | 11 | 0.35 |
| Cossidae | 15 | 0.29 | Asparagaceae | 18 | 0.56 |
| Crambidae | 171 | 3.32 | Asphodelaceae | 2 | 0.06 |
| Depressariidae | 129 | 2.50 | Aspleniaceae | 2 | 0.06 |
| Douglasiidae | 4 | 0.08 | Asteraceae | 319 | 10.01 |
| Drepanidae | 14 | 0.27 | Balsaminaceae | 4 | 0.13 |
| Elachistidae | 86 | 1.67 | Begoniaceae | 1 | 0.03 |
| Elaschistidae | 2 | 0.04 | Berberidaceae | 6 | 0.19 |
| Endromidae | 2 | 0.04 | Betulaceae | 35 | 1.10 |
| Epermeniidae | 21 | 0.41 | Bignoniaceae | 6 | 0.19 |
| Epicopeiidae | 1 | 0.02 | Blechnaceae | 1 | 0.03 |
| Erebidae | 235 | 4.56 | Boraginaceae | 41 | 1.29 |
| Eriocraniidae | 9 | 0.17 | Brassicaceae | 159 | 4.99 |
| Euteliidae | 2 | 0.04 | Bromeliaceae | 1 | 0.03 |
| Gelechiidae | 219 | 4.25 | Butomaceae | 1 | 0.03 |
| Geometridae | 659 | 12.79 | Buxaceae | 2 | 0.06 |
| Glyphipterigidae | 11 | 0.21 | Campanulaceae | 5 | 0.16 |
| Gracillariidae | 133 | 2.58 | Cannabaceae | 8 | 0.25 |
| Heliodinidae | 1 | 0.02 | Cannaceae | 1 | 0.03 |
| Heliozelidae | 18 | 0.35 | Capparaceae | 2 | 0.06 |
| Hepialidae | 7 | 0.14 | Caprifoliaceae | 44 | 1.38 |
| Hesperiidae | 63 | 1.22 | Caryophyllaceae | 65 | 2.04 |
| Heterogynidae | 12 | 0.23 | Casuarinaceae | 1 | 0.03 |
| Incurvariidae | 12 | 0.23 | Celastraceae | 7 | 0.22 |
| Lasiocampidae | 53 | 1.03 | Ceratophyllaceae | 1 | 0.03 |
| Limacodidae | 6 | 0.12 | Chlorophyta | 1 | 0.03 |
| Lycaenidae | 184 | 3.57 | Cistaceae | 33 | 1.04 |
| Lyonetiidae | 11 | 0.21 | Cleomaceae | 4 | 0.13 |
| Lypusidae | 3 | 0.06 | Colchicaceae | 1 | 0.03 |
| Micropterigidae | 10 | 0.19 | Combretaceae | 2 | 0.06 |
| Momphidae | 19 | 0.37 | Commelinaceae | 2 | 0.06 |
| Nepiculidae | 1 | 0.02 | Convolvulaceae | 21 | 0.66 |
| Nepticulidae | 253 | 4.91 | Coriariaceae | 1 | 0.03 |
| Noctuidae | 687 | 13.35 | Cornaceae | 4 | 0.13 |
| Nolidae | 30 | 0.58 | Crassulaceae | 22 | 0.69 |
| Notodontidae | 58 | 1.13 | Cucurbitaceae | 5 | 0.16 |
| Nymphalidae | 294 | 5.71 | Cupressaceae | 24 | 0.75 |
| Oecophoridae | 16 | 0.31 | Cycadaceae | 1 | 0.03 |
| Opostegidae | 6 | 0.12 | Cyperaceae | 44 | 1.38 |
| Papilionidae | 35 | 0.68 | Daphniphyllaceae | 2 | 0.06 |
| Pieridae | 81 | 1.57 | Dennstaedtiaceae | 2 | 0.06 |
| Plutellidae | 9 | 0.17 | Dicranaceae | 1 | 0.03 |
| Prodoxidae | 13 | 0.25 | Dilleniaceae | 1 | 0.03 |
| Psychidae | 14 | 0.27 | Dioscoreaceae | 2 | 0.06 |
| Pterolonchidae | 1 | 0.02 | Droseraceae | 2 | 0.06 |
| Pterophoridae | 148 | 2.87 | Dryopteridaceae | 4 | 0.13 |
| Pyralidae | 141 | 2.74 | Ebenaceae | 1 | 0.03 |
| Riodinidae | 1 | 0.02 | Elaeagnaceae | 5 | 0.16 |
| Roeslerstammiidae | 1 | 0.02 | Ephedraceae | 1 | 0.03 |
| Saturniidae | 20 | 0.39 | Equisetaceae | 1 | 0.03 |
| Schreckensteiniidae | 1 | 0.02 | Ericaceae | 37 | 1.16 |
| Scythrididae | 43 | 0.83 | Euphorbiaceae | 44 | 1.38 |
| Sesiidae | 128 | 2.48 | Fabaceae | 327 | 10.26 |
| Somabrachyidae | 1 | 0.02 | Fagaceae | 52 | 1.63 |
| Sphingidae | 72 | 1.40 | Frankeniaceae | 5 | 0.16 |
| Stathmopodidae | 1 | 0.02 | Gentianaceae | 16 | 0.50 |
| Thyrididae | 1 | 0.02 | Geraniaceae | 23 | 0.72 |
| Tineidae | 6 | 0.12 | Grossulariaceae | 7 | 0.22 |
| Tischeriidae | 4 | 0.08 | Haloragaceae | 2 | 0.06 |
| Tortricidae | 424 | 8.23 | Hydrangeaceae | 2 | 0.06 |
| Yponomeutidae | 69 | 1.34 | Hydrocharitaceae | 5 | 0.16 |
| Ypsolophidae | 16 | 0.31 | Hylocomiaceae | 1 | 0.03 |
| Zygaenidae | 59 | 1.14 | Hypericaceae | 23 | 0.72 |
|  |  |  | Hypnaceae | 1 | 0.03 |
|  |  |  | Iridaceae | 5 | 0.16 |
|  |  |  | Juglandaceae | 11 | 0.35 |
|  |  |  | Juncaceae | 16 | 0.50 |
|  |  |  | Juncaginaceae | 2 | 0.06 |
|  |  |  | Jungermanniaceae | 1 | 0.03 |
|  |  |  | Lamiaceae | 140 | 4.39 |
|  |  |  | Lardizabalaceae | 1 | 0.03 |
|  |  |  | Lauraceae | 3 | 0.09 |
|  |  |  | Lecythidaceae | 1 | 0.03 |
|  |  |  | Liliaceae | 5 | 0.16 |
|  |  |  | Linaceae | 7 | 0.22 |
|  |  |  | Loranthaceae | 1 | 0.03 |
|  |  |  | Lycopodiaceae | 1 | 0.03 |
|  |  |  | Lythraceae | 5 | 0.16 |
|  |  |  | Magnoliaceae | 3 | 0.09 |
|  |  |  | Malvaceae | 42 | 1.32 |
|  |  |  | Melanthiaceae | 3 | 0.09 |
|  |  |  | Menyanthaceae | 2 | 0.06 |
|  |  |  | Mniaceae | 1 | 0.03 |
|  |  |  | Moraceae | 6 | 0.19 |
|  |  |  | Musaceae | 1 | 0.03 |
|  |  |  | Myricaceae | 4 | 0.13 |
|  |  |  | Myrtaceae | 3 | 0.09 |
|  |  |  | Nyctaginaceae | 1 | 0.03 |
|  |  |  | Nymphaeaceae | 3 | 0.09 |
|  |  |  | Oleaceae | 42 | 1.32 |
|  |  |  | Onagraceae | 25 | 0.78 |
|  |  |  | Orchidaceae | 5 | 0.16 |
|  |  |  | Orobanchaceae | 20 | 0.63 |
|  |  |  | Oxalidaceae | 2 | 0.06 |
|  |  |  | Paeoniaceae | 1 | 0.03 |
|  |  |  | Papaveraceae | 24 | 0.75 |
|  |  |  | Parmeliaceae | 2 | 0.06 |
|  |  |  | Paulowniaceae | 2 | 0.06 |
|  |  |  | Pedaliaceae | 1 | 0.03 |
|  |  |  | Peltigeraceae | 2 | 0.06 |
|  |  |  | Phyllanthaceae | 1 | 0.03 |
|  |  |  | Phytolaccaceae | 1 | 0.03 |
|  |  |  | Pinaceae | 58 | 1.82 |
|  |  |  | Plantaginaceae | 53 | 1.66 |
|  |  |  | Platanaceae | 4 | 0.13 |
|  |  |  | Plumbaginaceae | 33 | 1.04 |
|  |  |  | Poaceae | 179 | 5.62 |
|  |  |  | Polygalaceae | 3 | 0.09 |
|  |  |  | Polygonaceae | 48 | 1.51 |
|  |  |  | Portulacaceae | 2 | 0.06 |
|  |  |  | Potamogetonaceae | 7 | 0.22 |
|  |  |  | Primulaceae | 19 | 0.60 |
|  |  |  | Proteaceae | 1 | 0.03 |
|  |  |  | Pteridaceae | 2 | 0.06 |
|  |  |  | Ranunculaceae | 42 | 1.32 |
|  |  |  | Resedaceae | 10 | 0.31 |
|  |  |  | Rhamnaceae | 29 | 0.91 |
|  |  |  | Rosaceae | 212 | 6.65 |
|  |  |  | Rubiaceae | 44 | 1.38 |
|  |  |  | Rutaceae | 21 | 0.66 |
|  |  |  | Salicaceae | 54 | 1.69 |
|  |  |  | Salvadoraceae | 1 | 0.03 |
|  |  |  | Salviniaceae | 1 | 0.03 |
|  |  |  | Santalaceae | 9 | 0.28 |
|  |  |  | Sapindaceae | 18 | 0.56 |
|  |  |  | Saxifragaceae | 17 | 0.53 |
|  |  |  | Scrophulariaceae | 15 | 0.47 |
|  |  |  | Simaroubaceae | 2 | 0.06 |
|  |  |  | Smilacaceae | 3 | 0.09 |
|  |  |  | Solanaceae | 37 | 1.16 |
|  |  |  | Sparganiaceae | 3 | 0.09 |
|  |  |  | Staphyleaceae | 1 | 0.03 |
|  |  |  | Styracaceae | 1 | 0.03 |
|  |  |  | Tamaricaceae | 8 | 0.25 |
|  |  |  | Taxaceae | 1 | 0.03 |
|  |  |  | Theaceae | 3 | 0.09 |
|  |  |  | Thymelaeaceae | 7 | 0.22 |
|  |  |  | Tropaeolaceae | 4 | 0.13 |
|  |  |  | Typhaceae | 4 | 0.13 |
|  |  |  | Ulmaceae | 9 | 0.28 |
|  |  |  | Urticaceae | 18 | 0.56 |
|  |  |  | Verbenaceae | 3 | 0.09 |
|  |  |  | Violaceae | 14 | 0.44 |
|  |  |  | Vitaceae | 15 | 0.47 |
|  |  |  | Zygophyllaceae | 5 | 0.16 |
